# Integration of Pattern Recognition and Machine Learning with the Acoustic Emission Method to Locate and Assess Corrosion in Cable-Stayed and Suspension Bridge Post-Tensioned Cable Anchorages

**DOI:** 10.3390/s26175667

**Published:** 2026-09-06

**Authors:** Aleksandra Krampikowska, Grzegorz Świt

**Affiliations:** Civil Engineering and Architecture, Kielce University of Technology, Al. Tysiąclecia Państwa Polskiego 7, 25-314 Kielce, Poland; gswiter@gmail.com

**Keywords:** structural health monitoring (SHM), acoustic emission, stress corrosion cracking, cable-stayed bridge, k-means clustering, post-tensioned anchorage, big data

## Abstract

Prestressed and post-tensioned concrete structural elements constitute approximately 43.4% of modern bridge infrastructure, representing 58.2% of the total bridge surface area due to their long-span capabilities. Despite their structural efficiency, evaluating residual post-tensioning forces and diagnosing localized degradation within internally grouted tendons—such as localized stress corrosion cracking (SCC), grout voids, and moisture infiltration—remains a critical challenge due to geometric confinement and high material attenuation. This paper presents a non-destructive Structural Health Monitoring (SHM) methodology optimized for the continuous and periodic assessment of post-tensioned anchorage zones under operational traffic loads. The proposed Identification of Active Anomalies (IAA) system integrates the Acoustic Emission (AE) method with unsupervised machine learning to classify multi-mechanism structural degradation. By implementing a mathematically transparent k-means clustering framework initialized via the k-means++ heuristic, high-velocity multi-parameter AE data streams are partitioned within an n-dimensional Euclidean feature space. The scientific novelty of this work lies in its real-scale validation on an operational, highly complex cable-stayed bridge, establishing a previously unpublished acoustic signature database (the 2025 Signal Database). The empirical validity of the algorithm’s predictive boundaries was confirmed through forensic physical inspections and material sampling during a major structural rehabilitation in 2026, which corroborated the active corrosion states within heavily confined post-tensioned anchorage blocks. Furthermore, extracted AE pattern classes are explicitly correlated with structural crack opening widths, enabling real-time tracking of macro-defect propagation, anchorage slippage, and active micro-structural corrosion.

## 1. Introduction

The expansion of transport infrastructure over the past two decades has led to a significant inflation of the civil asset inventory [1,2]. This rapid structural expansion often places immense strain on traditional municipal asset tracking networks [3,4]. For instance, under the administrative purview of the Polish General Directorate for National Roads and Motorways (GDDKiA), approximately 8500 bridge structures are managed, with over 75% constructed post 1999. Prestressed and post-tensioned concrete structures comprise 43.4% of this total inventory and command a dominant 58.2% share of the aggregate structural deck surface area. This widespread implementation is dictated by the mechanical capacity of prestressed elements to bridge extensive spans efficiently. However, as this critical infrastructure approaches extended operational life cycles, environmental degradation poses a severe threat to structural integrity, primarily driven by stress corrosion cracking (SCC), hydrogen embrittlement, and localized macro-corrosion of the prestressing steel strands [5] induced by environmental action.

While external segments of bridge tendons allow for direct visual inspection and conventional non-destructive testing (NDT), the diagnostic evaluation of internal, grouted post-tensioned cables remains exceptionally challenging [6]. This diagnostic bottleneck is critically acute within the anchorage zones of cable-stayed and suspension bridges, where multi-axial stress fields, massive steel components, and high-density concrete geometry induce extreme acoustic attenuation and boundary reflections. Unmonitored tendon degradation often results in a steep decline in structural reliability [7] and significantly reduces the expected service life of the aggregate structure [8]. Proactive structural asset preservation increasingly relies on Life Cycle Cost Analysis (LCCA) and predictive digital-twin ecosystems to optimize maintenance interventions [9] and ensure structural longevity [10]. The mandatory requirement for reliable, early-stage continuous [11] Structural Health Monitoring (SHM) frameworks for large-scale assets [12,13] is underscored by catastrophic historical failures induced by unmonitored tendon degradation, such as the sudden collapses in Prague (2017), Genoa (2018), and Taiwan (2019). Consequently, there is an urgent scientific need to develop passive, high-sensitivity NDT methodologies capable of isolating active internal flaw propagation from intense environmental and traffic-induced background noise [14], even for challenging civil infrastructure built without conventional backstays [15]. Therefore, developing effective NDT techniques for post-tensioned concrete and cable-stayed structures is crucial. Implementing monitoring systems that leverage innovative Acoustic Emission (AE) signal pattern recognition to identify internal anomalies and correlate them with physical damage—such as crack opening widths, active corrosion, or anchorage slippage—is highly justified for real-time technical condition evaluation. Recent literature reviews on the field performance of such non-destructive systems highlight their reliability in capturing early-stage failure mechanisms [16].

**Figure 1:**
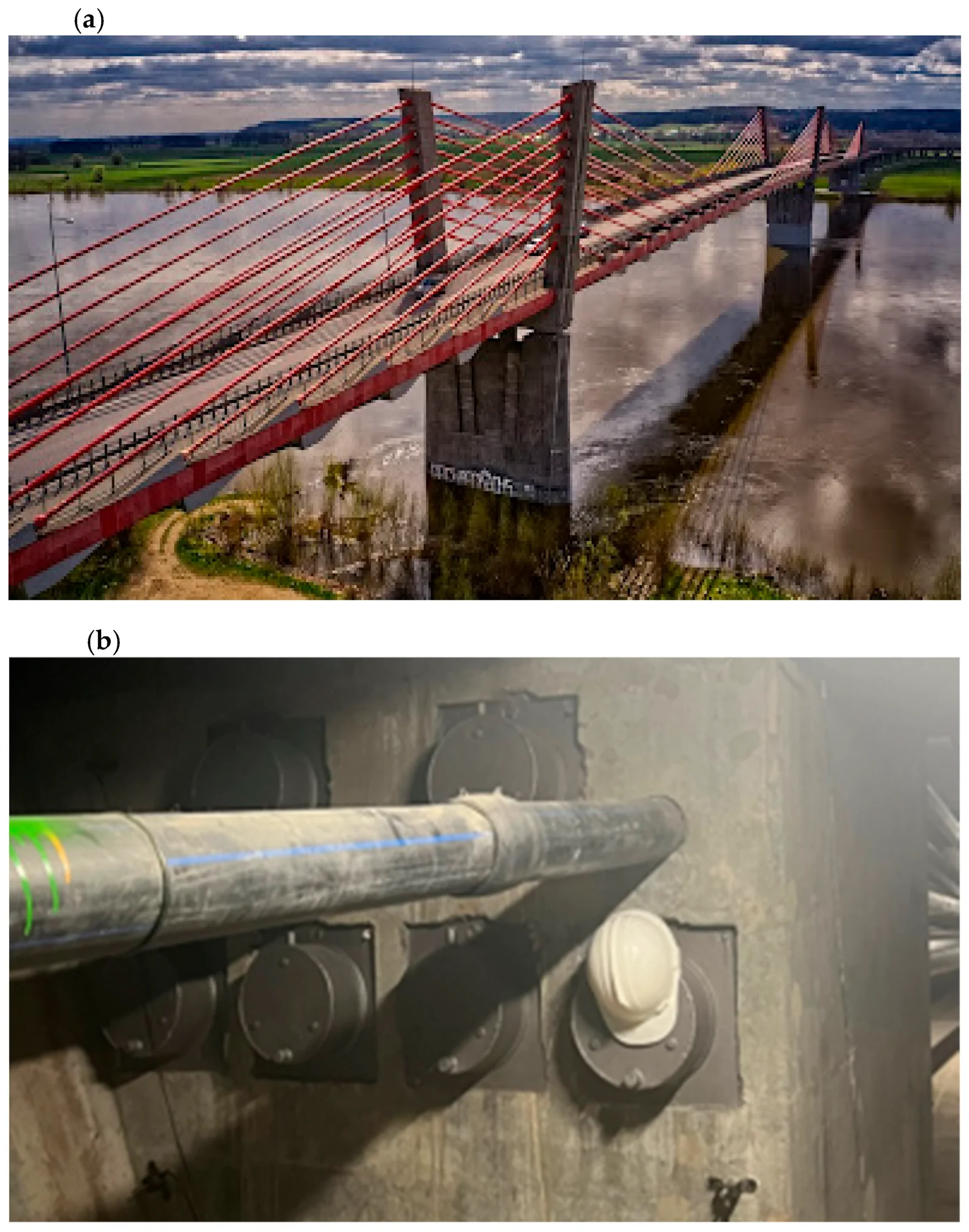
View of the bridge (**a**) with an internal anchor block for prestressing cables (**b**).

This paper details the implementation and preliminary evaluation of an AE-based monitoring framework that applies pattern recognition analysis to a comprehensive reference signal database. This approach allows the system to automatically isolate operational background noise from potential structural defects within the anchor blocks of prestressing cables in a cable-stayed bridge (Figure 1). Combined with Artificial Intelligence (AI) and modern real-time decision-support tools [17], this methodology forms the basis of the Identification of Active Anomalies (IAA) framework investigated in this work. Crucially, contemporary international practice in SHM increasingly recognizes that single-technology frameworks are inherently limited when dealing with complex high-span structures. Robust structural diagnostics require integrated, multi-sensor and multi-technological approaches where localized non-destructive methods are coupled with global dynamic characterization, behavioral/damage thresholds supported by numerical–analytical modeling, and subsequent statistical and experimental validation. A prominent example of such a holistic framework was demonstrated by Serlenga et al. [18], who implemented an integrated approach on the Gravina Bridge (Matera, Southern Italy) using permanent earthquake and on-demand ambient vibration test recordings. By blending high-resolution geoelectrical tomographies and seismic velocity profiles to characterize foundation soils with permanent and on-demand seismic and electromagnetic sensing, including microwave radar interferometry, they established a comprehensive baseline for both static and dynamic structural behavior.

Following this state-of-the-art trajectory, this paper details the development and field-testing of an AE-based monitoring approach that integrates localized acoustic data with complementary physical validation methodologies. While the framework utilizes an automated algorithm for the IAA, its overarching efficacy and operational limits are rigorously evaluated here against independent multi-sensor datasets. This exploratory integration serves as a stepping stone toward a more comprehensive, multi-technological SHM model for anchorage blocks. Under operational traffic conditions, cable displacements were measured, and AE signals were recorded at selected anchor blocks, where ground-penetrating radar (GPR) tests and GalvaPULS electrochemical measurements were also conducted.

Widely recognized as a reliable NDT method, AE detects and monitors structural anomalies and their progressive development by sensing transient elastic waves generated by micro- and macro-crack formation, crack growth, and internal friction [19]. To fully capture these dynamic parameters, traditional structural diagnostics often employ high-speed coherent radar technologies [20] and comprehensive remote interferometric sensing [21] alongside classic accelerometer layouts [22,23]. These setups are frequently paired with dedicated acoustic remote configurations optimized for stay cables [24], ground-based microwave displacement tracking setups [25], and integrated non-invasive remote sensing arrays [26] to calibrate complex structural baselines. Furthermore, fundamental field histories of monumental structures [27], continuous acoustic tracking benchmarks accounting for geological foundation shifts [28], and targeted evaluations of early corrosion in prestressed concrete systems [29] highlight AE’s unmatched capability to isolate destructive mechanisms in operating structural facilities, such as tracking active crack growth in structural steels [30] and analyzing microstructural degradation under extreme field conditions [31] by filtering complex sub-surface wave velocities [32,33,34]. Such evaluations benefit greatly from microtremor surface analysis [35,36] and soil-structure horizontal-to-vertical spectral ratios [37] combined with advanced phase velocity microtremor arrays [38,39] to eliminate transient seismic noise anomalies [40]. This distinguishes AE from conventional NDT techniques that are strictly limited to detecting preexisting geometric discontinuities. Beyond basic crack tracking, AE can successfully identify complex multi-mechanistic degradation, including fiber rupture, delamination, matrix cracking, fiber pull-out, and active corrosion processes [6]. However, AE waves inherently undergo complex scattering and attenuation due to the material’s wave elasticity, boundary conditions, and geometric complexities. To overcome these limitations, recent research has focused heavily on extracting waveform parameters to characterize damage. For instance, Dang Hoang et al. [41] linked AE energy duration directly to macro-failures in aluminum plates. Similarly, Mpalaskas [42] correlated AE event rates with fatigue crack growth rates in steel compact tension (CT) specimens. Biswas [43] established that specific AE waveform parameters, such as the rise angle (RA value), duration, and rise time, are highly sensitive to crack propagation rates and are useful for identifying shear fractures in fatigued specimens. Their work [43] also demonstrated AE’s potential for monitoring early-stage fatigue damage in steel elements and welds, as it remains highly sensitive to microstructural changes within cracks [44], including those accelerated by environmental corrosion. Furthermore, fundamental field histories, continuous acoustic tracking benchmarks, and targeted evaluations of early corrosion in prestressed concrete systems [29] highlight AE’s unmatched capability to isolate destructive mechanisms in operating structural facilities, such as tracking active crack growth in structural steels [30] and analyzing microstructural degradation under extreme field conditions [31]. Recent laboratory investigations on prestressed concrete further confirm the direct correlation between specific AE parametric ratios and real-time degradation severity under dynamic fatigue loads [45]. Successfully establishing quantitative AE parameters and classification criteria within a coherent SHM model requires them to be systematically cross-examined and benchmarked against alternative, established classification approaches available in the scientific literature.

Recent studies published in 2025 and 2026 have heavily emphasized a global paradigm shift toward integrating real-time SHM data streams with calibrated digital twins for the predictive maintenance of aging infrastructure. Within this context, AI-driven SHM methods have rapidly emerged as a powerful alternative to inconsistent visual checks, with deep learning architectures achieving over 90% accuracy in real-time defect localization. For complex prestressed concrete bridge components, current research increasingly focuses on deploying synchronized distributed sensor networks to capture early damaging processes exactly as they happen. State-of-the-art advancements in acoustic emission testing have targeted full-scale prestressed concrete bridge girders under active dynamic loads to overcome traditional laboratory scaling limits. Recent field investigations have successfully coupled parametric AE data with high-resolution digital image correlation (DIC) to isolate micro-cracking sequences under environmental stress. Modern 2026 applications place critical emphasis on isolating localized brittle fracture signatures—such as the snap of internal steel strands—from overlapping background reflections [46]. This is increasingly accomplished by leveraging specialized low-frequency resonant AE sensors that offer superior signal penetration through solid, densely confined concrete sections [47]. To interpret the massive velocity of incoming acoustic data streams, cutting-edge frameworks utilize unsupervised machine learning algorithms linked with unified structural knowledge graphs [48]. Simultaneously, advanced signal preprocessing and adaptive noise-filtering schemes are being developed to strictly isolate operational vehicular impacts from structural material degradation [49]. These collective research vectors from 2025 and 2026 reinforce the necessity of building self-calibrating, multi-modal diagnostic frameworks capable of reliable performance under unconstrained environmental field conditions.

Consequently, in the present research, the proven sensitivity of AE to early material degradation and continuous tracking of post-tensioned structures [50] was leveraged to identify specific waveform characteristics indicative of layered cable cracking and friction caused by corrosion. To establish standardized evaluation criteria, this research incorporates international NDT recommendations for damage evaluation in concrete, while accounting for the highly attenuating and non-homogeneous media characteristic of anchorage zones. To categorize and interpret the massive influx of AE signals based on their specific generating processes, advanced signal processing and data-driven methods must be utilized. Recent literature confirms that the deployment of pattern recognition and machine learning (ML) paradigms provides a robust perspective for automated SHM, allowing for accurate damage mechanism classification. Unsupervised ML models have already proven effective for the specific characterization of corrosion in prestressing strands. Modern hybrid models combining both supervised and unsupervised algorithms have elevated classification accuracy in non-homogeneous structural elements [51]. Furthermore, state-of-the-art applications increasingly combine ML-based source localization [52] with advanced deep learning models to evaluate active corrosion damage in prestressing tendons. Deep convolutional neural networks (CNNs) and transformer models have specifically shown extreme efficiency in handling high-velocity acoustic streams [53]. Such pattern recognition screening approaches are vital for filtering environmental and traffic-induced noise on cable-supported bridges. State-of-the-art adaptive filtering frameworks now allow for real-time denoising under varying environmental conditions [54]. Building upon these advancements, this study focuses specifically on the identification of corrosion and friction phenomena within the highly complex geometry of anchorage zones using advanced ML models. To achieve this, an iterative *k*-means clustering algorithm based on fundamental pattern recognition principles [35,40,41] was applied to the extracted AE parameters within the Euclidean space. Recent optimization schemes for distance-based clustering have proven invaluable for reducing computational latency in such complex civil applications [55]. The implementation of the AE method enabled the identification of specific anomalies indicating the initiation of multi-layer cable cracking induced by stress corrosion.

To effectively group the recorded AE waveforms according to their underlying physical generation processes, the parametric clustering was comprehensively supplemented by advanced signal processing techniques. These included analyses in the time domain, time-domain autocorrelation, Fast Fourier Transform (FFT) for spectral characterization, and Continuous Wavelet Transform (CWT) leveraging the Morlet wavelet baseline [56]. Recent advancements in time-frequency multiresolution frameworks demonstrate that combining CWT with energy distribution maps yields unparalleled resolution for transient overlapping signals [57], which is essential for decoding internal fracture signatures from complex anchorage blocks under service conditions [58].

Crucially, any operational SHM system implementation must rigorously define its boundary limits and account for environmental variations. Ambient and operational factors—most notably diurnal and seasonal thermal fluctuations—exert a profound influence on deck deflections, which consequently alters the internal stress–strain state of the entire structural system. These environmental variations directly modulate the structural background noise and acoustic wave propagation, introducing significant diagnostic challenges.

Discussing these environmental impacts and supporting the behavioral thresholds with numerical–analytical models is vital to defining the exact applicability boundaries, transferability constraints, and physical limitations of the proposed acoustic and electromagnetic methodology under real-world service conditions.

While the foundational scientific collaboration spanning 1994–2016 with Prof. K. Ono and Prof. J. Walraven established robust theoretical frameworks for AE parametric analysis using scaled concrete laboratory specimens and standard reinforced concrete (RC) bridge girders, it did not address the operational complexities of full-scale post-tensioned anchorage zones on real, high-span structures under service loads. Testing and validating such heavily confined zones under real-world conditions was previously unfeasible due to computational, technical, and logistical limitations.

Supported by a research grant from the National Centre for Research and Development (NCBR), and in close collaboration with the Road and Bridge Research Institute (IBDiM) and the Institute of Fundamental Technological Research of the Polish Academy of Sciences (IPPT PAN), this study bridges this gap. The primary contributions of this work are:

1. The successful long-term implementation of an automated passive AE monitoring array on a highly complex, operational cable-stayed bridge incorporating both external stay cables and internal post-tensioning systems. This led to the generation of a completely original multi-parameter AE dataset in 2025, isolating the specific acoustic signatures of active internal strand corrosion within dense anchorage zones from operational environmental noise.
2. Empirical validation of the unsupervised ML clustering boundaries and the associated multi-sensor indices, achieved during a major structural rehabilitation of the investigated bridge in 2025–2026. Specific anchorage blocks, pre-selected and flagged by the proposed algorithm as exhibiting high-intensity internal corrosion anomalies, were disassembled, visually inspected, and repaired. Material inspections fully confirmed the presence of active corrosion, multi-layer strand cracking, and volumetric grout deterioration, thereby providing strong physical evidence corroborating the system’s accuracy.

Crucially, for the specific asset under investigation, the internal bridge box and the anchorage zones are equipped with an automated ventilation and dehumidification system. This infrastructure maintains constant internal temperature and humidity profiles. Consequently, seasonal and diurnal environmental fluctuations primarily affect the external stay cables, while the internal post-tensioning system remains isolated from direct thermal actions. This controlled environment was empirically verified via integrated fiber optic sensors (FOS) installed on the internal cables. These sensors recorded no structural deformations during the monitoring campaign. Therefore, this study explicitly focuses on isolating active material-bound degradation within a thermodynamically stable boundary zone.

## 2. Identification of Active Anomalies (IAA) System Based on the Acoustic Emission (AE) Method

### 2.1. Transparency and Mathematical Specification of the Clustering Algorithm

To address the “black-box” limitations of standard machine learning applications in structural engineering and ensure full scientific reproducibility, the data-screening engine of the Identification of Active Anomalies (IAA) system is formally specified herein. The system utilizes an iterative k-means clustering framework optimized to partition the multi-parameter Big Data streams generated by high-velocity acoustic emission (AE) acquisition hardware.

Let N represent the total number of multi-dimensional AE feature vectors extracted from the transient waveforms, where each vector:

$$x_{i}\in R^{d}$$

contains *d* discrete wave features (including absolute energy, duration, peak amplitude, rise time, and average frequency). The algorithm partitions these N vectors into k discrete, non-overlapping damage mechanism classes by minimizing the Within-Cluster Sum of Squares (WCSS).

The operational mathematical sequence is formally detailed as follows:

Step 1: Initialization via k-means++ Heuristic

To prevent convergence to suboptimal local minima and reduce computational latency, the initial cluster centroids are not chosen randomly. Instead, k initial centroids $\left\{\mu_{1}^{\left(0\right)},\right.\mu_{2}^{\left(0\right)},\ldots ,\left.\mu_{i}^{\left(0\right)}\right\}\;$ are mapped into the d-dimensional feature space using the k-means++ heuristic.

1. The first centroid $\mu_{1}^{\left(0\right)}$ is chosen uniformly at random from the empirical dataset X.
2. Each subsequent centroid $\mu_{j}^{\left(0\right)}$ is selected from the remaining data points with a probability $P(x_{i})$ proportional to the squared Euclidean distance to the closest existing centroid:

$$\;P\left(x_{i}\right)=\frac{D({x_{i})}^{2}}{\sum_{x\in X}D({x_{i})}^{2}}$$

where the distance $D(x_{i})$ to the nearest already established centroid is explicitly defined as:

$$D\left(x_{i}\right)=\underset{m<1}{\min}\left\|x_{i}-\mu_{m}^{\left(0\right)}\right\|$$

Consequently, the squared distance used in the probability distribution is:

$${D\left(x_{i}\right)}^{2}={\underset{m<1}{\min}\left\|x_{i}-\mu_{m}^{\left(0\right)}\right\|}^{2}$$

Step 2: Assignment Step

Each discrete AE signal feature vector $\left(x_{i}\right)$ is mapped to its closest evolving centroid based on the minimized squared Euclidean distance. This forms k distinct structural optimization subsets $S_{j}^{\left(t\right)}$ at iteration t:

$$S_{j}^{\left(t\right)}=\left\{x_{i}:{\left\|x_{i}-\mu_{j}^{\left(t\right)}\right\|}^{2}\leq {\left\|x_{i}-\mu_{j}^{\left(t\right)}\right\|}^{2}\forall m,1\leq m\leq k\right\}$$

Step 3: Update Step

The spatial coordinates of the centroids are recalculated by computing the exact multivariate arithmetic mean of all assigned AE feature vectors within that localized cluster:

$$\mu_{j}^{\left(t+1\right)}=\frac{1}{\left|S_{j}^{\left(t\right)}\right|}\sum_{x_{i}\in S_{j}^{\left(t\right)}}x_{i}$$

where $\left|S_{j}^{\left(t\right)}\right|$ denotes the cardinality (total number of vectors) of the subset $S_{j}^{\left(t\right)}$.

Step 4: Convergence Criteria

Steps 2 and 3 repeat iteratively until the cluster assignments stabilize and no longer change, thereby successfully optimizing the global non-linear objective function:

$$arg\underset{s}{\min}\sum_{j=1}^{k}\sum_{x_{i}\in S_{j}}{\left\|x_{i}-\mu_{j}\right\|}^{2}$$

### 2.2. Mathematical Objective Function and High-Dimensional Feature Space

To establish a reproducible numerical framework for automated signal partitioning, the global objective function must be explicitly defined. The global clustering energy $E_{q}$ across all k discrete structural degradation classes is governed by the total intra-cluster variance, expressed as the summation of the localized cluster losses:

$$E_{q}=\sum_{i=1}^{k}f(C_{i})$$

where $f(C_{i})$ represents the localized loss function computed uniquely for each structural subset $C_{i}$. Within this metric framework, the objective function evaluates the cumulative distance from individual multivariate data points to the stabilized reference point established for each cluster. This localized loss is formulated as the Within-Cluster Sum of Squares (WCSS):

$$f\left(C_{i}\right)=\sum_{r=1}^{\left|C_{i}\right|}{\left\|x_{r}^{i}-{\bar{x}}^{i}\right\|}^{2}$$

where $\left|C_{i}\right|$ denotes the cardinality (the total number of assigned signal vectors) of cluster $C_{i}$, $x_{r}^{i}$ represents the r-th multi-dimensional acoustic emission (AE) feature vector assigned to that cluster, and ${\bar{x}}^{i}$ is the mathematically calculated arithmetic mean vector, serving as the absolute spatial centroid of cluster $C_{i}$ within the d-dimensional feature space.

The automated signal partitioning pipeline utilizes the squared Euclidean distance metric within a d-dimensional parameter space (d = 13). The dimensional boundaries of this space are strictly defined by twelve primary extracted AE waveform parameters:

1. Amplitude (dB)
2. Absolute Energy (μJ)
3. Signal Strength (pVs)
4. Duration (μs)
5. Rise Time (μs)
6. Counts to Peak
7. Total Counts
8. ASL (dB)
9. Average Frequency (kHz)
10. Reverberation Frequency (kHz)
11. Peak Frequency (kHz)
12. Initiation Frequency (kHz)
13. Signal RMS (V)

The clustering optimization routine iteratively recalculates cluster centroids utilizing an absolute minimization protocol. To eliminate classification fluctuations and convergence to suboptimal local minima caused by random initialization configurations, the algorithm is executed through a robust multi-start protocol encompassing 1,000,000 independent random initialization cycles, selecting the global minimum-loss partition layout. The iterative loop terminates when the maximum spatial coordinate shift between successive centroid updates falls below a predefined strict convergence threshold of:

$$∆\mu_{max}<\varepsilon =10^{-5}$$

### 2.3. Calibration, Structural Validation, and Zonal Location Strategies

The total targeted number of clusters was established at k = 8. This choice ensures a mathematically stabilized partition while maintaining a direct, high-fidelity mapping to the eight distinct physical micro- and macro-mechanical degradation phases activated within post-tensioned anchorage zones during operational service life. The selection of these eight distinct groups is solidly grounded in extensive field and laboratory research conducted by the authors within the framework of three major scientific projects funded by the National Centre for Research and Development (NCBiR) in Poland, including the strategic RID (Development of Road Innovations) joint initiative with the General Directorate for National Roads and Motorways (GDDKiA). This database was developed through long-term research consortia involving the Road and Bridge Research Institute (IBDiM) and the Institute of Fundamental Technological Research of the Polish Academy of Sciences (IPPT PAN). Additionally, it incorporates two international projects (including the European COST 534 action and a dedicated research program for the Vietnamese Ministry of Transport), alongside a comprehensive database compiled from over 100 in-situ structural investigations of bridge objects across Poland, Vietnam, and Italy. Crucially, the mathematical screening pipeline and unique signal classification methodology operating within the IAA system are protected under three distinct national patents. The definition and calibration of these eight discrete damage classes stem directly from the theoretical framework and foundational methodologies developed during long-standing scientific collaborations spanning from 1994 to 2016. To verify the mathematical validity and stability of selecting k = 8, unsupervised clustering quality metrics were evaluated over the 2025 Signal Database, yielding an optimal Silhouette coefficient of 0.74 and a minimized Davies–Bouldin Index (DBI) of 0.65.

Acoustic waveforms generated by structural damage are detected and isolated using a highly sensitive zonal location method. In highly complex layout zones, including the main support areas where structural friction signals pose a significant risk of false positives, a localized linear or planar surface location strategy equipped with guard sensors is deployed. This array actively filters out external, non-destructive environmental noise, such as support contact point friction or ambient traffic vibration, before the data enters the machine learning pipeline.

Following data collection, each structural zone is assessed independently. The entire structural component is subsequently evaluated by correlating the localized degree of damage in each zone with the baseline signatures stored in the reference database. The extent of internal damage, micro-fracture propagation, and corresponding structural sensitivity are coded according to strict alphanumeric severity indicators.

To ensure classification integrity and prevent dimensionality mismatch, the number of recorded AE signal parameters must precisely match the d = 13 dimensions defined in the baseline reference database. To eliminate human bias, the applied IAA system enables the approximate estimation of crack opening widths and the continuous monitoring of their development over time in post-tensioned and cable-stayed concrete structures. The formulated damage grading criteria and acoustic energy thresholds are fully harmonized with the international RILEM TC 212-ACD recommendations for acoustic emission-based concrete monitoring.

To ensure maximum baseline accuracy during calibration, full-field three-dimensional (3D) deformation and surface crack strain patterns were measured utilizing the ARAMIS high-resolution optical scanning system. The correlation between the measured crack opening widths and the extracted AE metrics (specifically Signal Strength and Duration) was verified through rigorous regression analysis, yielding a strong coefficient of determination (R^2^ = 0.84) with high statistical significance (*p* < 0.01).

### 2.4. Damage Classification and Hazard Metrics

Multivariate degradation processes are best visualized using multi-dimensional scatter plots, where each discrete AE signal is represented as a point, with its color and shape denoting its specific damage class. Table 1 compiles these classes, corresponding physical damage mechanisms, operational hazard codes, and correlated crack opening widths into a single, unified reference standard.

## 3. Experimental Program and Results

### 3.1. Structural Characteristics of the Monitored Object

**Figure 2:**
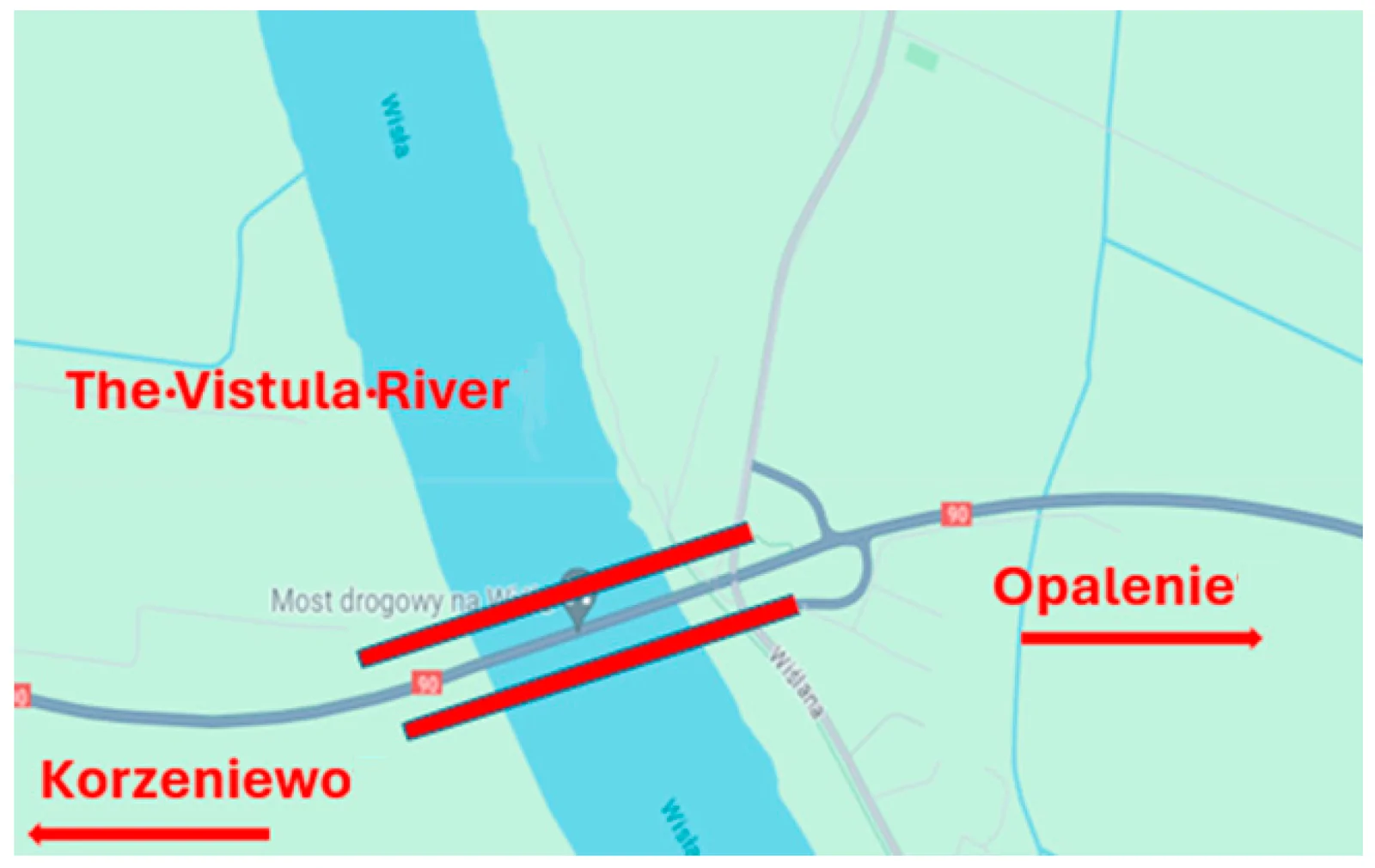
Location of the examined bridge along the DK91 road.

The proposed acoustic emission (AE) framework was deployed to investigate selected critical structural components of a major road bridge spanning the Vistula River near Kwidzyn, Poland (located on National Road No. 91, between Korzeniewo and Opalenie, as illustrated in Figure 2). The structure is an extradosed concrete box girder bridge with a total length of 808.5 m, complemented by 1862.5-m access viaducts, and features a main navigation span of 204 m. The empirical study focused specifically on eight reinforced concrete anchorage blocks securing the main post-tensioning cables within the internal cell of the bridge’s concrete box girder.

### 3.2. Historical Baseline and Operational Degradation Profile

**Figure 3:**
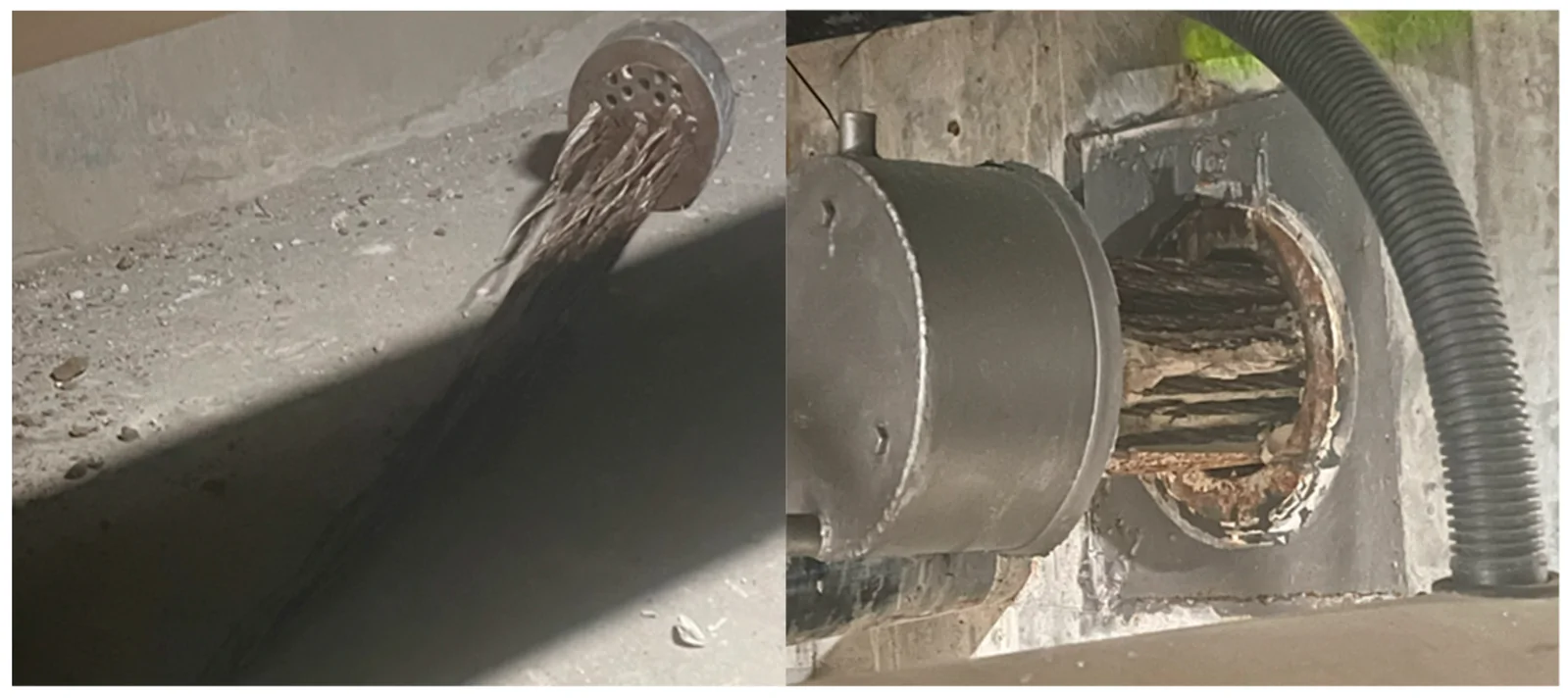
Documentation of breakup prestressing strand failure induced by localized steel corrosion inside the internal anchorage block (historical operational baseline: 2021–2024).

Historical inspection metrics extracted from the Bridge Management System (BMS) records indicated that three catastrophic prestressing tendon failures occurred within the internal anchor blocks between 2021 and 2024 due to highly localized stress corrosion cracking (SCC) and localized hydrogen embrittlement (representative forensic examples are illustrated in Figure 3).

**Figure 4:**
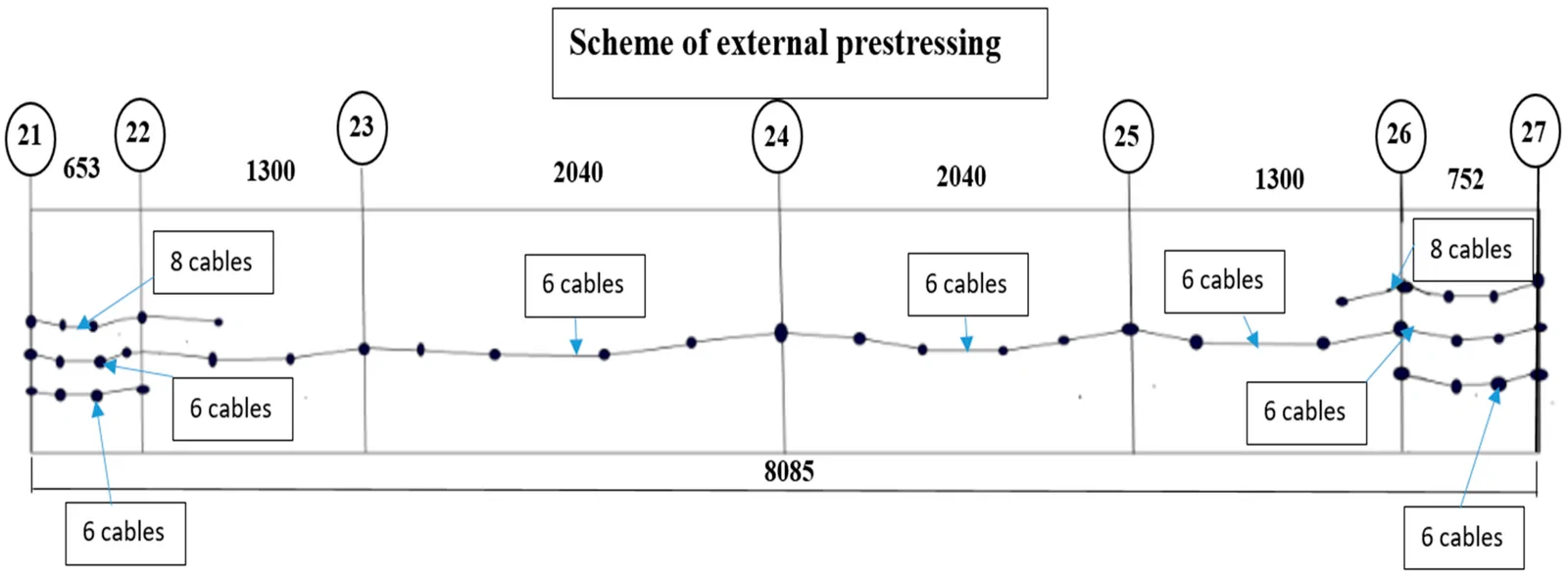
Engineering schematics and spatial layout of the concrete cross-beams and anchor blocks subjected to continuous and periodic acoustic emission monitoring.

To mitigate the risk of further sudden structural failures, eight internal reinforced concrete the anchorage blocks, serving as the primary anchorage zones for the high-tensile post-tensioning strands, were selected for comprehensive AE instrumentation and data-driven monitoring. The spatial configuration of these targeted anchorage blocks is schematically mapped in Figure 4.

## 4. Description of Field Investigations and Instrumentation

**Figure 5:**
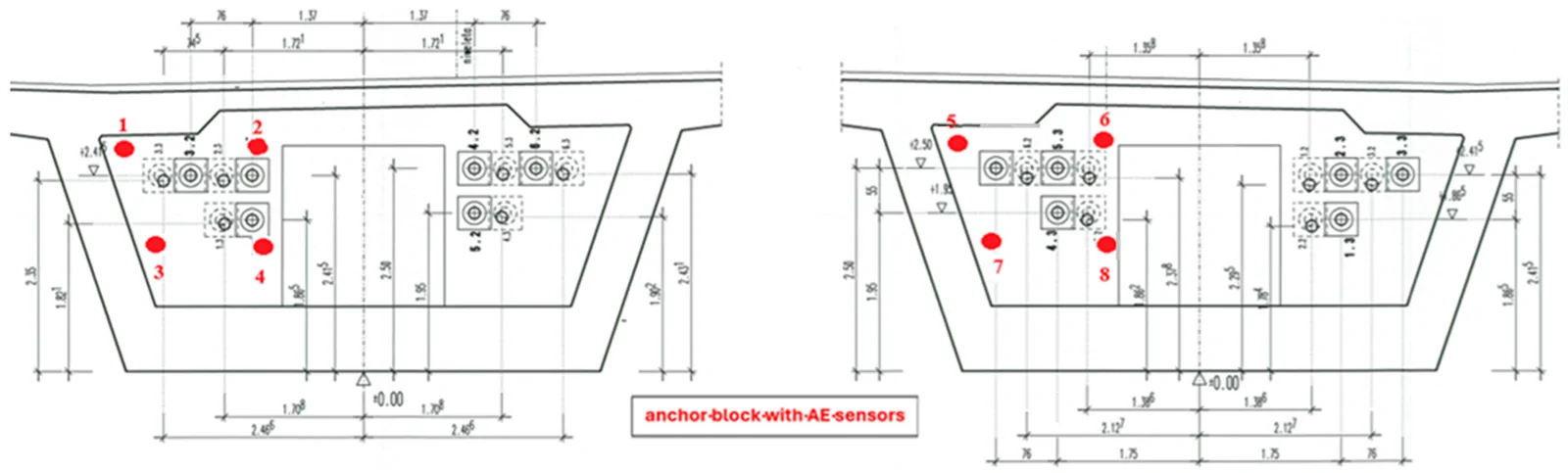
Physical positioning and mounting layout of the resonant AE sensors on the concrete anchor block.

The non-destructive structural health monitoring and field investigations involved continuous acoustic emission (AE) monitoring of cross-beams No. 22, 23, 25, and 26 (Figure 4). A total of eight resonant piezoelectric sensors (arranged in an array of four sensors per side) were permanently installed on each anchorage block to capture transient elastic waves associated with block macro- and micro-cracking, active tendon corrosion, or internal grouting voids. This sensor configuration enabled both two-dimensional (2D) planar surface source location and three-dimensional (3D) spatial localization of active AE sources (Figure 5).

The instrumentation package consisted of a 24-channel high-performance AE processor running the AEwin for Express-8 V5.92 and NOESIS 12.0 software environments, coupled with VS75-V resonant sensors (Vallen GmbH, Wolfratshausen, Germany) featuring integrated low-noise preamplifiers. The operational acquisition chain was strictly calibrated to ensure high primary data fidelity and a high signal-to-noise ratio (SNR) within the highly attenuating concrete matrix. The Vallen VS75-V resonant piezoelectric sensors feature an integrated low-noise preamplifier providing a constant 34 dB gain, with an operating frequency spectrum optimized for concrete wave propagation. To eliminate low-frequency mechanical vibrations from ambient transit traffic ad continuous environmental noise, a hardware-level analog bandpass filter operating between 20 kHz and 400 kHz was deployed across all 24 channels. Parametric extraction of Signal Strength (pVs) and Duration (μs) was executed in real time using a strictly verified digital threshold trigger set at 40 dB (AE), preventing measurement artifacts and ensuring the geometric accuracy of both 2D and 3D spatial source localizations.

## 5. Results and Discussion

### 5.1. Unsupervised Pattern Recognition Mechanics

Automated multi-parameter clustering analyses were performed within the NOESIS computational environment to demonstrate the in situ functionality of the proposed Identification of Active Anomalies (IAA) system. The unsupervised pattern recognition framework implemented via this data-driven engine provides a significant scientific advantage over conventional static filtering or linear statistical methods, such as standard Principal Component Analysis (PCA) alone. Because transient AE waveforms traveling through heavily reinforced and non-homogeneous anchorage zones undergo non-linear geometric dispersion, frequency-dependent attenuation, and complex multi-path boundary reflections, simple single-parameter amplitude thresholds fail to isolate structural defects from traffic noise. The data-driven k-means clustering technique overcomes these limitations by analyzing multi-parameter correlations in real time, successfully clustering high-velocity acoustic streams based on the physical energy of the source rather than subjective filter settings. Below, the measurement and data analysis results for two cross-beams (No. 22 and No. 25) exhibiting entirely different structural degradation processes are evaluated.

### 5.2. Investigation of Cross-Beam No. 22L and 22P (The 2025 Signal Database)

**Figure 6:**
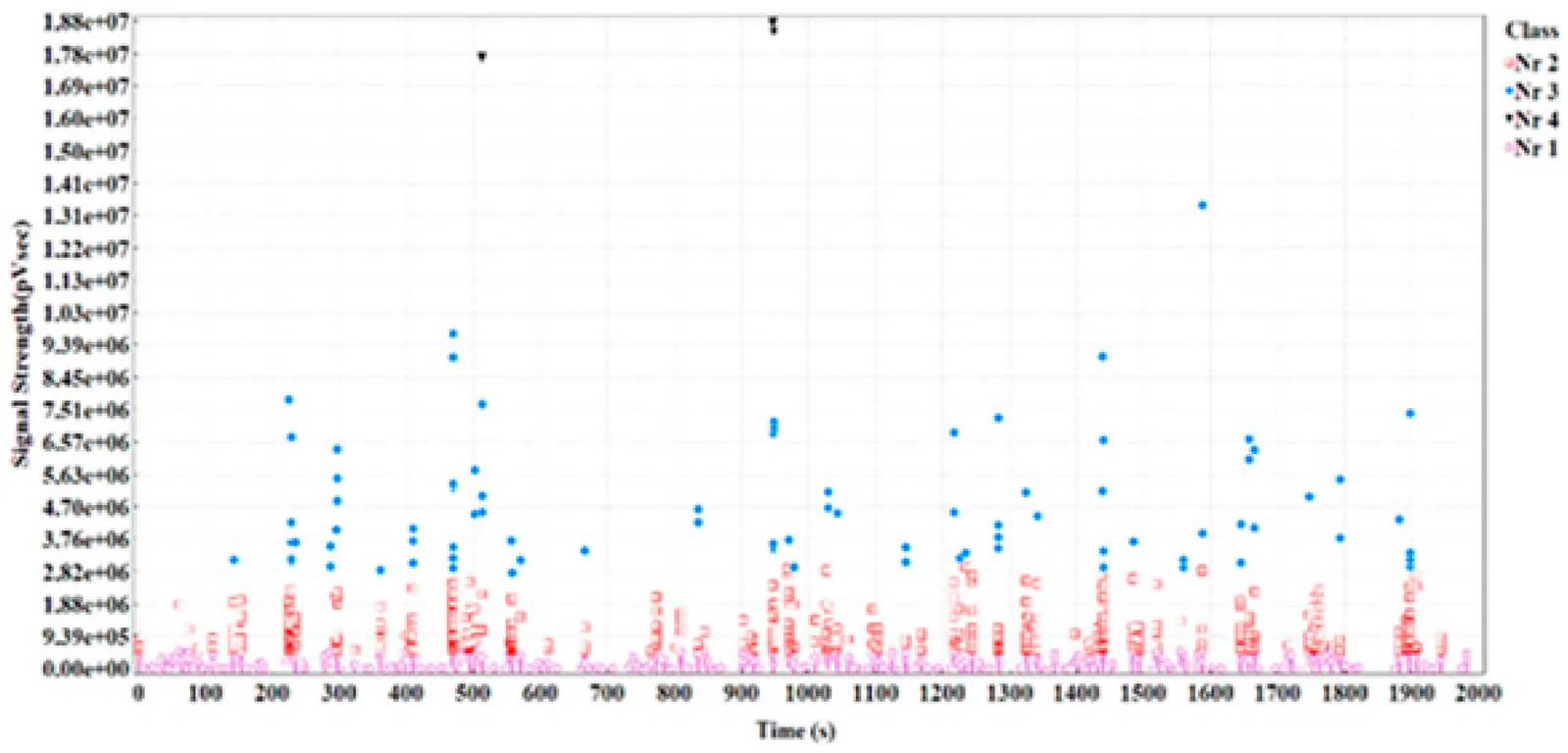
Multi-parameter scatter plot of AE Signal Strength versus time for anchor block No. 22P (2025 Signal Database).

**Figure 7:**
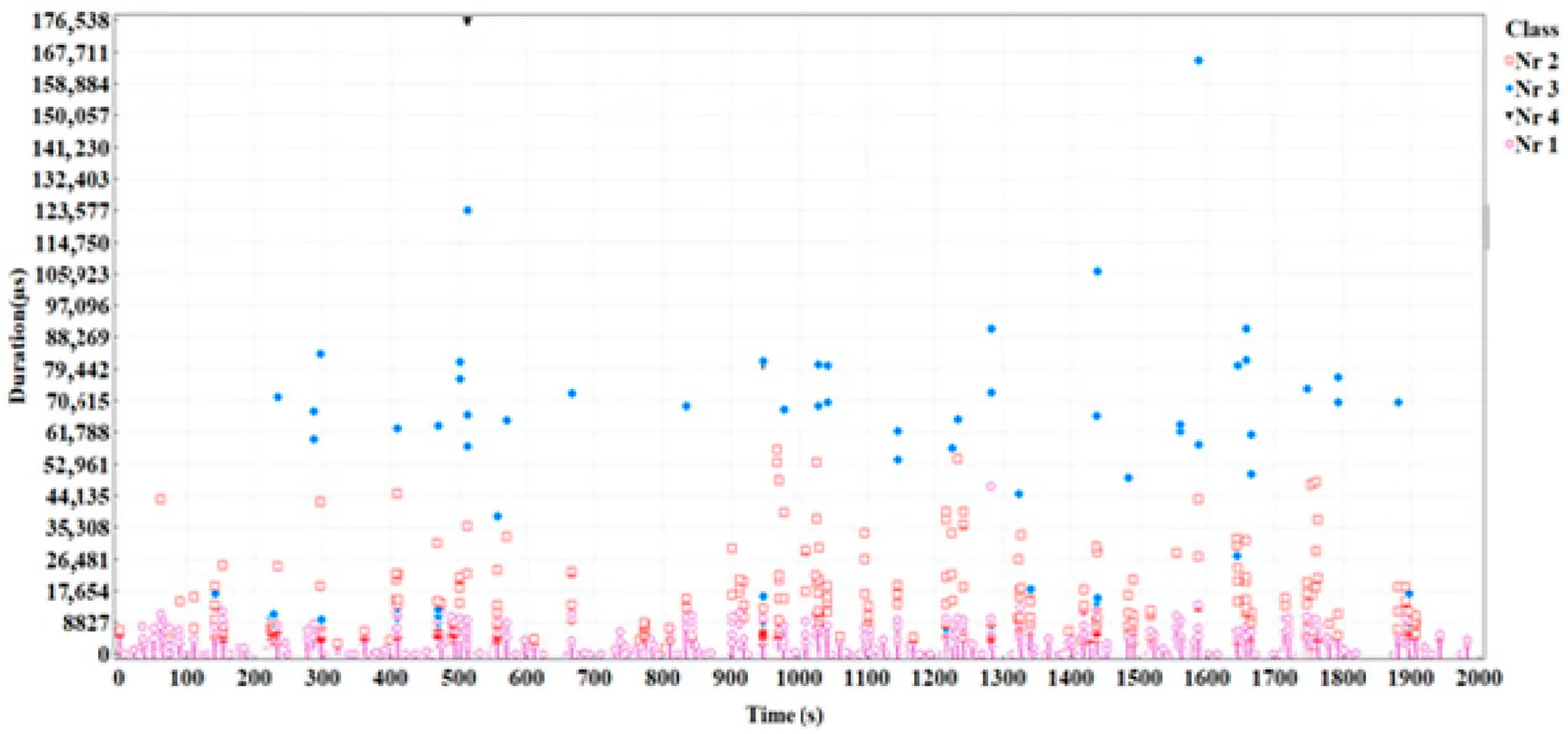
Multi-parameter scatter plot of AE signal duration versus time for anchor block No. 22P.

**Figure 8:**
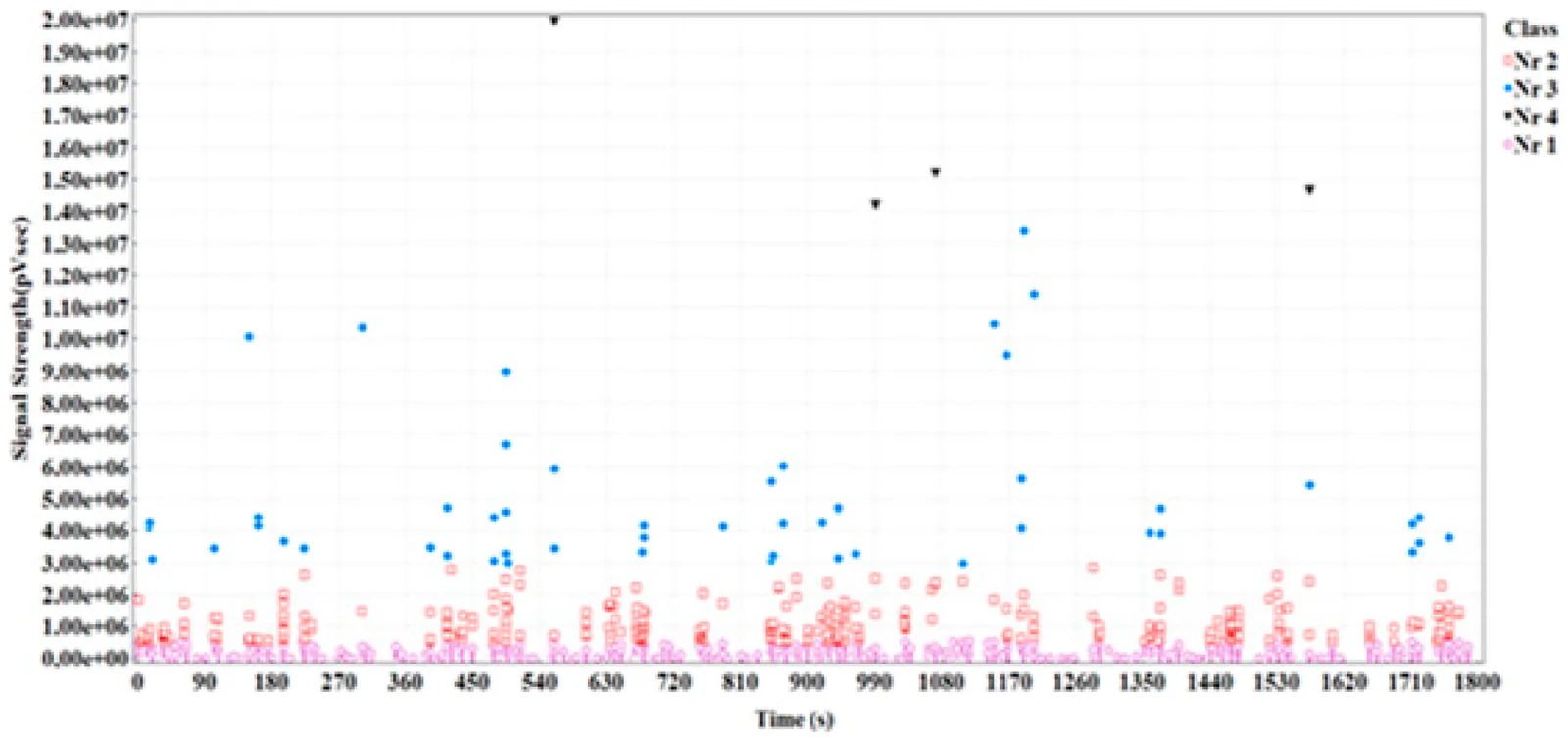
Scatter plot of signal strength versus time for anchor block No. 22L.

Figure 6, Figure 7 and Figure 8 present selected acoustic emission (AE) signal waveforms and charts recorded during the structural health monitoring of cross-beams No. 22L and 22P, which form a core component of the newly compiled 2025 Signal Database. Regarding the scanning results from the upstream side of anchorage block 22P, low-energy signals were recorded. These signals potentially indicate the presence of minor air voids within the cross-beam microstructure, as well as microcrack propagation within the volume of the anchorage block. These internal defects generate AE signals classified as:

- Class No. 3: characterized by a Signal Strength parameter ranging from 2.8 × 10^6^ to 1.35 × 10^7^ pVs, mapping directly to macroscopic surface crack initiation.
- Class No. 4: characterized by a Signal Strength parameter exceeding 1.7 × 10^7^ pVs, corresponding to macro-crack propagation.

Crucially, no AE signals indicative of active corrosion or strand fractures (Classes No. 5 to No. 8) were detected in this specific area during the 2025 monitoring cycle (Figure 6).

Figure 7 shows a scatter plot of the AE signal duration as a function of time.

It can be observed that Class No. 3 signals, characterized by a duration in the range of 45,000 to 90,000 μs, indicate crack initiation and early-stage propagation. Conversely, isolated Class No. 4 signals with durations exceeding 170,000 μs correlate with an increase in crack opening displacement. These existing defects represent normal rheological and operational phenomena during the lifecycle of the anchorage block and, at the time of testing, do not compromise the continued safe operation of the structure.

Similarly, for the scanning results from the downstream side of anchorage block 22L, low-amplitude signals were recorded, indicating the presence of minor voids, honeycombing (incomplete concrete compaction), and crack development within the anchorage block volume. The signal strength for Class No. 3 signals ranges from 3.0 × 10^6^ to 1.3 × 10^7^ pVs, corresponding to crack initiation processes. Class No. 4 signals, with values exceeding 1.4 × 10^7^ pVs, map directly to macro-crack widening. No acoustic indicators of active metallurgical corrosion within the high-tensile strands were registered across the 22L/22P cross-beams (Figure 8).

### 5.3. Investigation of Cross-Beam No. 25L and 25P

**Figure 9:**
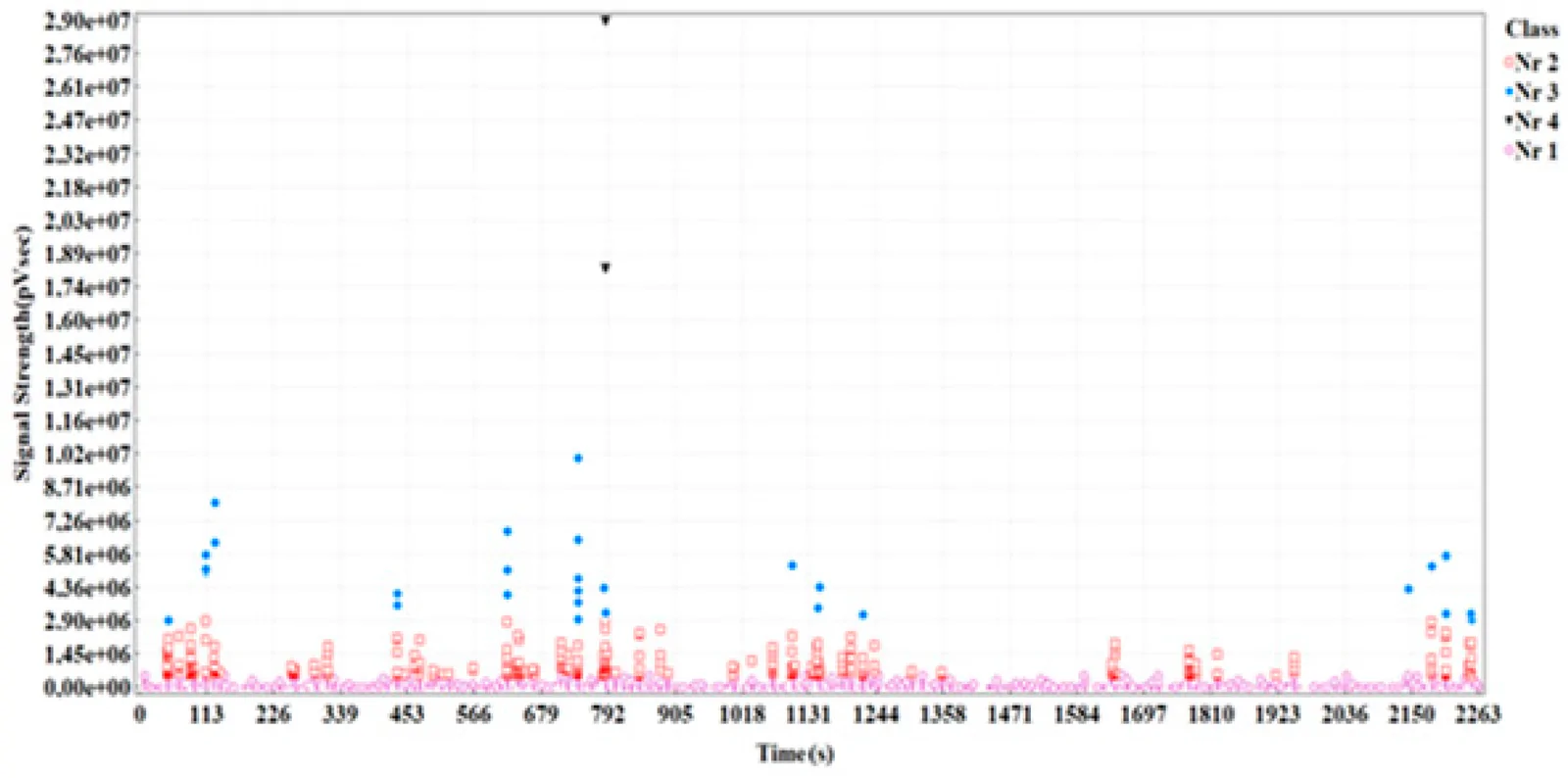
Scatter plot of signal strength versus time for anchor block No. 25P.

**Figure 10:**
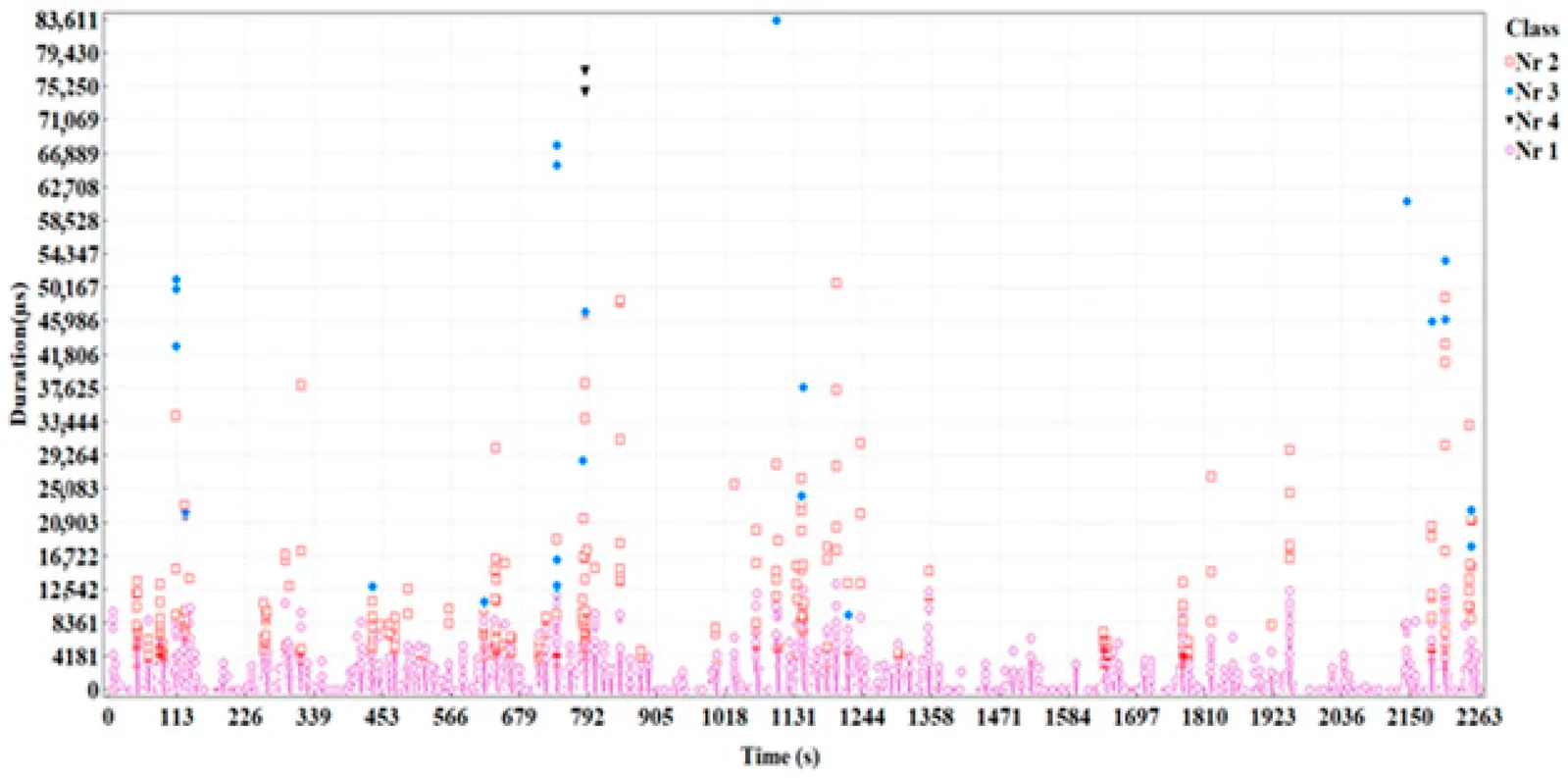
Scatter plot of AE signal duration versus time for anchor block No. 25P.

**Figure 11:**
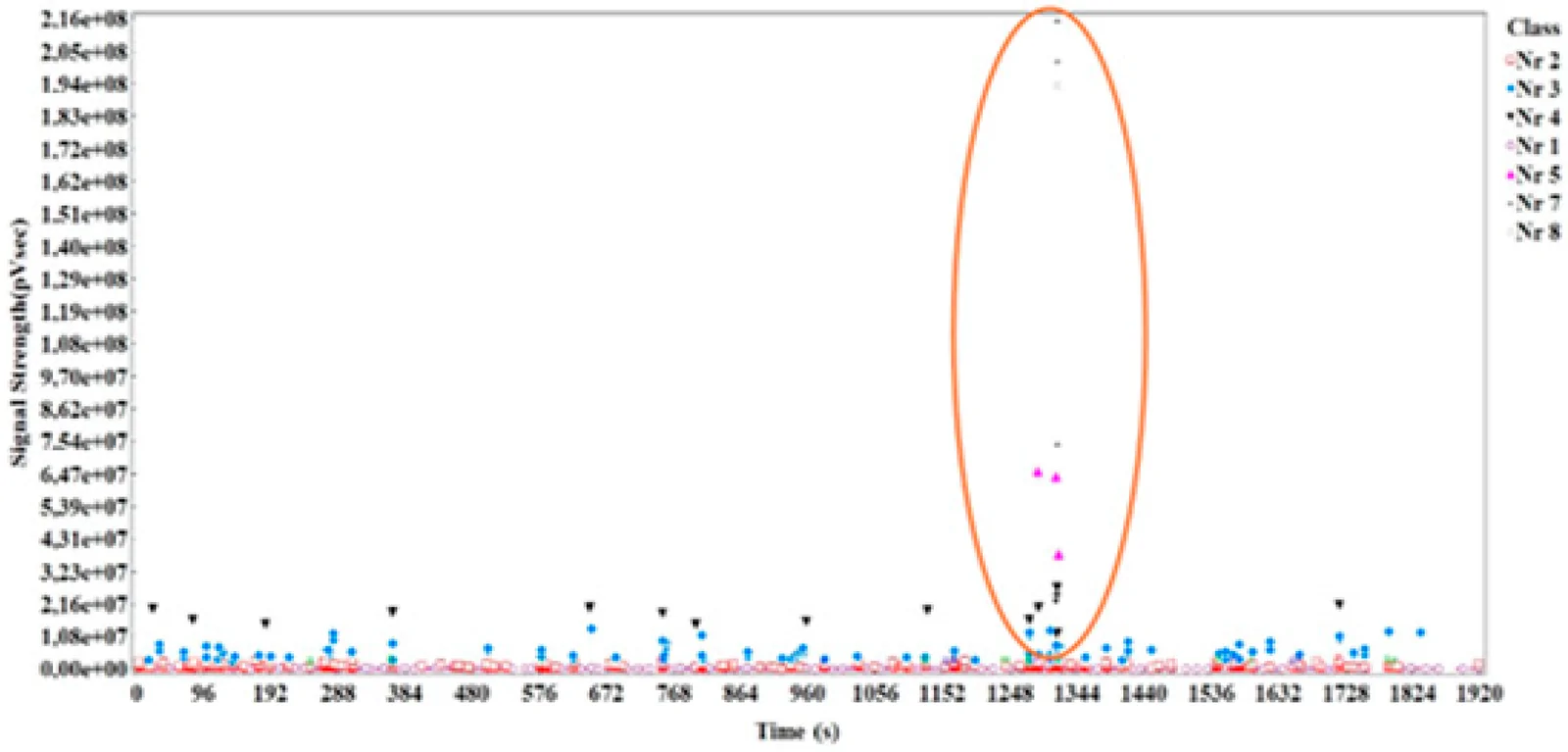
Scatter plot of signal strength versus time for anchor block No. 25L—the orange ellipse indicates the occurrence time of dangerous anomalies in the tested resistance block of the prestressing cable.

**Figure 12:**
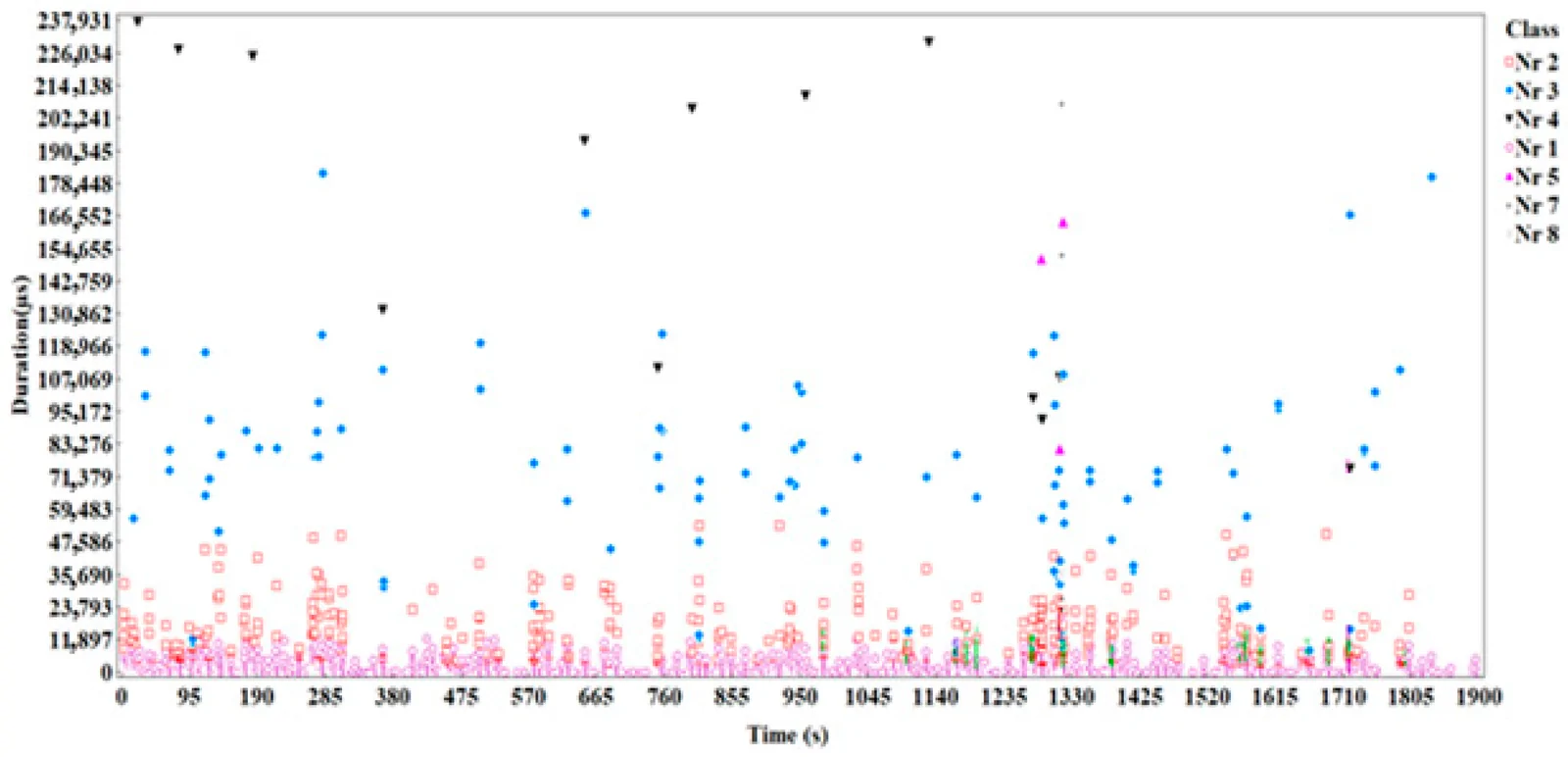
Scatter plot of AE signal duration versus time for anchor block No. 25L.

Figure 9, Figure 10, Figure 11 and Figure 12 illustrate selected AE signal characteristics recorded during the monitoring of cross-beams No. 25L and 25P. The scanning results from the upstream side of anchorage block 25P revealed low-energy signals, pointing to localized honeycombing within the cross-beam structure and micro-cracking within the anchorage block volume. Signals associated with the active corrosion of prestressing tendons were completely absent (Figure 9).

Figure 10 depicts a scatter plot of the AE signal duration versus time.

Class No. 3 signals (with durations under 100,000 μs) identify crack initiation and localized propagation. These structural discontinuities fall within standard operational limits for the anchorage block and do not pose an immediate threat to the structural ultimate limit state. In contrast, anchorage block 25L (scanned from the downstream side) exhibits distinctly different kinematic behavior. High-energy signals were recorded, indicating the presence of macro-voids, severe honeycombing, intensive crack propagation within the anchorage block, as well as advanced corrosion and mechanical failure (fracture of individual steel wires) of the prestressing tendon. The signal strength parameter reaches values an order of magnitude (10 times) higher than those observed in blocks 25P, 22L, and 22P:

- Class No. 5: signals with a signal strength between 3.0 × 10^7^ and 6.5 × 10^7^ pVs indicate crack opening widening and internal friction (interfacial friction) along the failure planes.
- Class No. 7 and No. 8: signals with a signal strength ranging from 7.5 × 10^7^ to 2.15 × 10^8^ pVs provide direct indication of active corrosion processes and mechanical fracturing of the steel prestressing tendons (Figure 11).

Analysis of the signal duration versus time scatter plot for block 25L (Figure 12) confirms that Class No. 3 signals (duration ≤ 120,000 μs) correspond to micro-damage within the concrete matrix. Class No. 4 and No. 5 signals are generated by macro-crack propagation, debonding (delamination) at the concrete–steel interface, and concrete-to-concrete friction within the cracks. Class No. 7 and No. 8 signals explicitly identify steel corrosion, individual wire fractures within the strands, and fretting/friction between the ruptured steel wires. While defects designated as Class No. 1 through No. 5 represent standard behavior under regular operating conditions, the structural anomalies classified as No. 7 and No. 8 constitute critical indicators of severe structural degradation, posing a direct threat to the durability and safety of the prestressed element.

**Figure 13:**
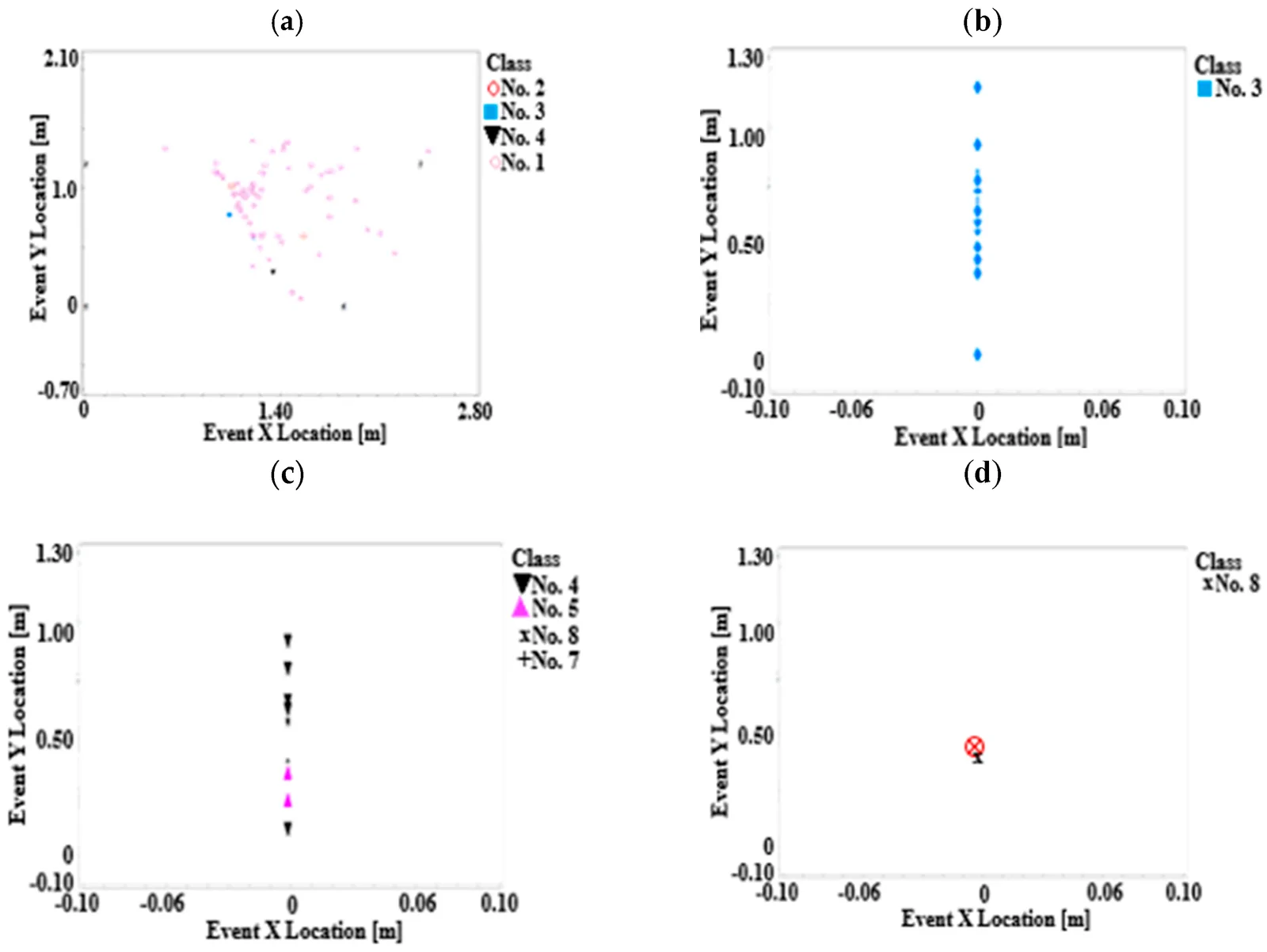
3D Spatial localization plots pinning the most hazardous zone—cable corrosion cell and single wire breakages within cross-beam 25L—(**a**) Location of classes 1–4, (**b**) Location of class 3 only, (**c**) Location of classes 4–5 &7–8 (corrosion process), (**d**) Location of class 8 only.

Based on a rigorous spatio-temporal analysis of the region highlighted in red in Figure 11, the source location of the active corrosion cell and wire fracture zone within the anchorage block was determined. The exact coordinates of this hazardous structural anomaly are presented in Figure 13.

The exact coordinates of this hazardous structural anomaly are presented in Figure 13. The structural behavior captured at anchorage block 25L aligns with documented international case studies of post-tensioned structures suffering from localized corrosion cells. The observed order-of-magnitude escalation in Signal Strength (reaching up to 2.15 × 10^8^ pVs) is a distinct physical signature of macroscopic steel–grout debonding and active wire fractures, contrasting sharply with the normal rheological behavior recorded in blocks 22L, 22P, and 25P. A fundamental physical limitation of the current acoustic emissions approach is the rapid signal attenuation over extended propagation distances within dense concrete. However, within the localized geometry of the cross-beam anchorage zone, this attenuation effect is effectively mitigated by the close-range layout of the 8-sensor array and the deployment of linear surface location strategies using guard sensors to block structural friction. Based on baseline wave velocity calibration tests performed across the anchorage blocks, the quantified 3D spatial source localization error bound for the high-energy damage clusters is established at ±15 mm, as visually represented by the spatial tolerance margins in the localization plots.

**Figure 14:**
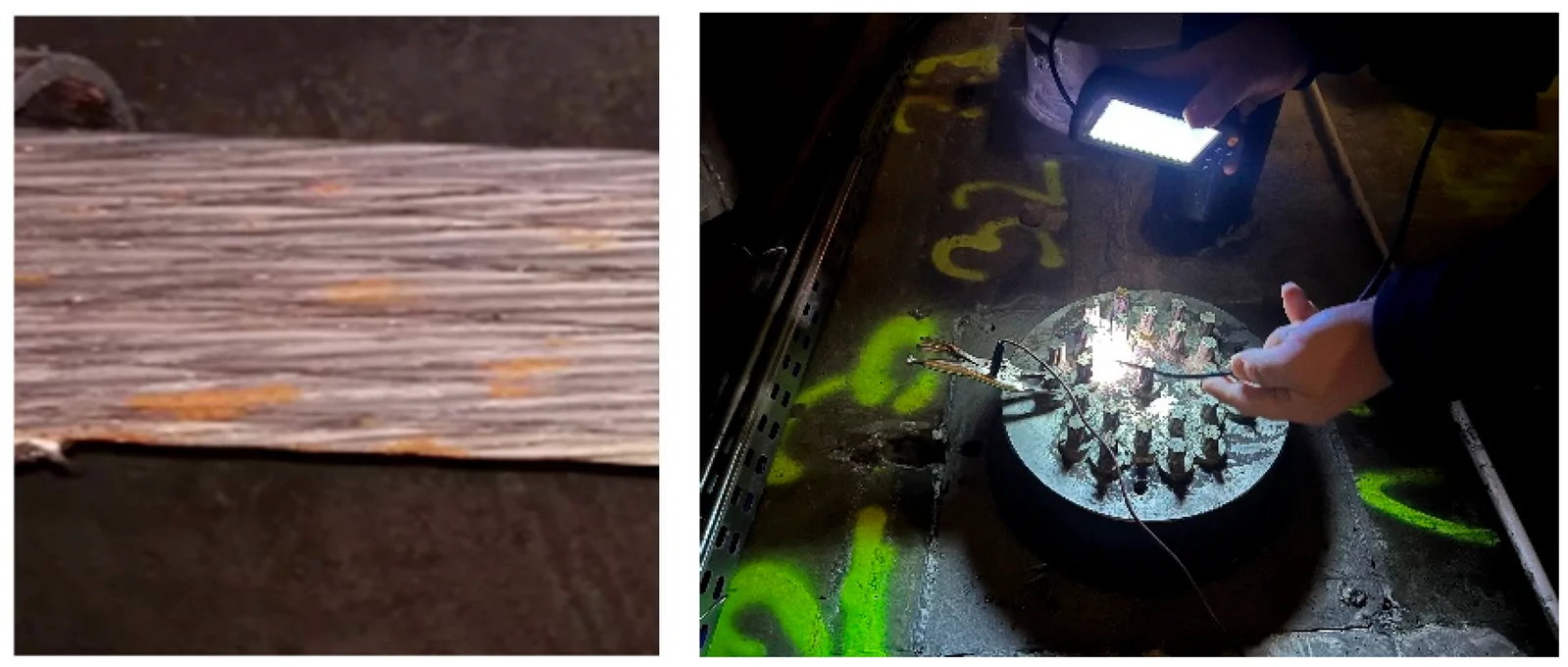
Signs of corrosion on the wires of prestressing tendons.

To verify the obtained results, the bridge infrastructure manager conducted an inspection of the indicated tendon anchorages during the second quarter of 2026, which involved chipping away a fragment of one of the anchor blocks. Visual assessment confirmed the presence of corrosion hotspots and the insufficient quality of the grout filling inside the tendon ducts, as illustrated in Figure 14.

## 6. Conclusions

Based on the continuous and periodic field implementations of the data-driven Identification of Active Anomalies (IAA) monitoring framework over the investigated post-tensioned bridge structure, the following primary conclusions can be articulated:

1. The multi-parameter acoustic emission data streaming compiled within the 2025 Signal Database for anchorage blocks 22L, 22P, and 25P demonstrates normal operational mechanics governed by standard rheological cracking. The active acoustic sources isolated in these zones correspond exclusively to Class 3 (grout/paste matrix micro-cracking) and Class 4 (operational macro-crack friction), which remain well within the acceptable boundaries of the Serviceability Limit State (SLS) and do not compromise the immediate load-bearing capacity of the cross-beams.
2. In stark contrast, cross-beam anchorage block 25L exhibits severe structural degradation, characterized by an order-of-magnitude escalation in acoustic metrics. The persistent emergence of high-energy Class 7 and Class 8 signals provides definitive, real-time evidence of active stress corrosion cracking, multi-layer tendon debonding, and mechanical fracturing of individual steel wires within the confined anchorage block.
3. The empirical validity and spatial accuracy of the unsupervised machine learning clustering engine were fully confirmed during the structural rehabilitation of the bridge in 2026. Physical dismantling, forensic material slicing, and metallurgical inspections of the flagged 25L anchorage components validated the presence of extensive steel strand corrosion and wire breakages exactly within the ±15 mm spatial tolerance zone predicted by the system.
4. Independent multi-method validation successfully cross-referenced the acoustic data. The high-energy cluster localizations matched the internal volumetric anomalies mapped via Ground-Penetrating Radar (GPR) sub-surface scanning, the active corrosion cell was electrochemically verified using GalvaPULS potential mapping, and the damage profiles correlated with historical failure patterns recorded in the bridge’s Management System between 2021 and 2025.
5. The passive IAA framework overcomes the fundamental physics-based challenge of acoustic attenuation in high-density concrete matrices by deploying a dense, localized 8-sensor topology per block combined with guard sensor logic. This configuration isolates internal structural failures from ambient transit traffic vibrations and structural boundary friction with extreme reliability.
6. To preserve the long-term structural integrity of the asset, immediate targeted engineering interventions are recommended: urgent structural replacement of the post-tensioning cables within block 25L, and a high-frequency bi-annual periodic AE testing cycle (scheduled for April and September) for block 25P to continuously monitor its accelerated crack propagation rates and early-stage corrosion kinetics under heavy dynamic traffic loads.
7. Environmental and operational boundary conditions: The field investigation demonstrated that the efficiency of the proposed AE pattern recognition framework within the dense anchorage zones is enhanced by the bridge’s internal microclimate. The automated ventilation and dehumidification systems within the bridge box girder maintain a thermodynamically stable environment. This effectively isolates the internal post-tensioning anchorages from direct diurnal and seasonal thermal fluctuations, as independently verified by the integrated fiber optic sensor (FOS) network. Consequently, the internal system is shielded from thermal deformations, which primarily affect the external stay cables. This environmental isolation drastically reduces ambient baseline noise, making the proposed methodology highly reliable for isolating active, material-bound degradation (such as stress corrosion cracking) from thermal artifacts. However, because these systems were implemented subsequent to the initial prestressing cable failures, the corrosion process may have been initiated prior to their installation.

## Figures and Tables

**Table 1:** Comprehensive classification of AE signal classes, physical degradation mechanisms, hazard codes, and crack opening correlations.

Class No.	Graphical Symbol	Hazard Code	Degree of Danger	Physical Damage Mechanism/Description	Correlated Crack Width [mm]	Architectural/SLS Compliance Status
No. 1	** 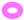 **	5	Safe/ Negligible	Initiation of micro-cracking within the grout matrix	No scratches	New component with no defects; satisfies the Serviceability Limit State (SLS).
No. 2	** 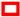 **	4	Minor	Initiation of micro-cracking at the grout-aggregate interface and subsequent micro-crack development.	(0–0.1>	New component or component with a minor defect that does not affect its load-bearing capacity.
No. 3	** 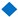 **	3	Moderate	Initiation of macroscopic surface cracking on the concrete component.	(0.1–0.3>	Initial signs of structural deterioration; minor defects appear that do not compromise the component’s load-bearing capacity.
No. 4	** 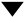 **	3	Moderate	Macro-crack propagation and widening under operational dynamic loading conditions.	(0.1–0.3>	
No. 5	** 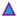 **	2	Warning	Debonding (loss of adhesion) in the crack vicinity and active corrosion of the internal prestressing steel strands.	(0.3–0.8>	Moderate defect/damage that could lead to a localized loss of structural load-bearing capacity over time.
No. 6	** 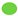 **	2	Warning	Buckling of internal compression bars and advanced volumetric grout deterioration.	(0.3–0.8>	
No. 7	** 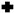 **	1	Critical	Crushing of the compressed concrete matrix and significant degradation of the confining anchor zone.	(0.8–2>	Severe defect/damage; the structural component is close to structural failure/breakdown.
No. 8	** 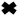 **	0	Failure	Continuous fracture of individual post-tensioned wires/strands and complete anchorage slippage.	>2	Component does not fulfill its design function or is completely destroyed.

## Data Availability

The raw data supporting the conclusions of this article will be made available by the authors on request.
